# The roles of AIM2 in neurodegenerative diseases: insights and therapeutic implications

**DOI:** 10.3389/fimmu.2024.1441385

**Published:** 2024-07-15

**Authors:** Kai Yang, Xi Wang, Hanyu Pan, Xinqing Wang, Yunhan Hu, Yihe Yao, Xinyue Zhao, Taolei Sun

**Affiliations:** ^1^ School of Chemistry, Chemical Engineering and Life Science, Wuhan University of Technology, Wuhan, China; ^2^ Hubei Key Laboratory of Nanomedicine for Neurodegenerative Diseases, School of Chemistry, Chemical Engineering and Life Science, Wuhan University of Technology, Wuhan, China; ^3^ Hunan Key Laboratory of Typical Environmental Pollution and Health Hazards, School of Public Health, Hengyang Medical School, University of South China, Hengyang, China; ^4^ Institute of WUT-AMU, Wuhan University of Technology, Wuhan, China; ^5^ State Key Laboratory of Advanced Technology for Materials Synthesis and Processing, Wuhan University of Technology, Wuhan, China

**Keywords:** inflammasome, AIM2, neurodegenerative disease, Alzheimer’s disease, Parkinson’s disease

## Abstract

AIM2, a cytosolic innate immune receptor, has the capability to recognize double-stranded DNA (dsDNA). This paper delineates the structural features of AIM2 and its mechanisms of activation, emphasizing its capacity to detect cytosolic DNA and initiate inflammasome assembly. Additionally, we explore the diverse functions of AIM2 in different cells. Insights into AIM2-mediated neuroinflammation provide a foundation for investigating novel therapeutic strategies targeting AIM2 signaling pathways. Furthermore, we present a comprehensive review of the roles of AIM2 in neurodegenerative diseases, including Alzheimer’s disease (AD) and Parkinson’s disease (PD). Finally, we discuss its therapeutic implications. In conclusion, a profound understanding of AIM2 in neurodegenerative diseases may facilitate the development of effective interventions to mitigate neuronal damage and slow disease progression.

## 1 Introduction

Neurodegenerative diseases are characterized by the progressive degeneration of neurons in the central nervous system (CNS), resulting in cognitive decline and/or motor dysfunction. These disorders present significant challenges to global healthcare systems and impose a substantial economic burden on society. Prominent examples of neurodegenerative diseases include Alzheimer’s disease (AD), Parkinson’s disease (PD) and amyotrophic lateral sclerosis (ALS). The etiology of neurodegenerative diseases is multifactorial, involving complex interactions between genetic and environmental factors. While genetic mutations predispose individuals to certain neurodegenerative diseases, environmental toxins and lifestyle factors also contribute to disease onset and progression (1).

Emerging evidence suggests neuroinflammation, including both innate and acquired immunity, plays crucial roles in the pathophysiology of these diseases, contributing to the initiation and progression of neurodegeneration (2–4). The innate immune system provides the first line of defense, primarily through the actions of microglia, the resident immune cells of the CNS (5). Meanwhile, the acquired immune system, involving adaptive immune responses, adds another layer of complexity to the immune dynamics in neurodegenerative conditions (6).

Innate immunity in the CNS is largely mediated by microglia, which are activated in response to neuronal injury, pathogens, and aggregated proteins commonly found in neurodegenerative diseases. In AD, microglia recognize amyloid-beta (Aβ) plaques through pattern recognition receptors (PRR) such as toll-like receptors (TLRs) and nucleotide-binding oligomerization domain (NOD)-like receptors (NLRs) (7). Upon activation, microglia initiate a cascade of pro-inflammatory responses, releasing cytokines, chemokines, and reactive oxygen species (ROS). While these responses aim to clear pathological proteins and debris, chronic activation can lead to sustained inflammation and neuronal damage (8). Similarly, in PD, microglia become activated in response to α-synuclein aggregates, contributing to the progressive loss of dopaminergic neurons in the substantia nigra (9).

Astrocytes, another key player in the CNS innate immune response, also become reactive in neurodegenerative diseases. Reactive astrocytes undergo morphological and functional changes, including the upregulation of glial fibrillary acidic protein (GFAP) and the release of pro-inflammatory mediators (10). In neurodegenerative diseases such as ALS, reactive astrocytes contribute to motor neuron degeneration by releasing toxic substances and exacerbating inflammatory responses. The interplay between microglia and astrocytes amplifies the neuroinflammatory environment, promoting further neuronal injury (11).

The acquired immune system, comprising T cells and B cells, also significantly impacts neurodegenerative disease progression. In the context of neurodegenerative diseases like AD and PD, there is increasing evidence of T cell infiltration into the CNS. These T cells can recognize and respond to neuronal antigens, contributing to the inflammatory milieu (6). For example, in AD, the presence of Aβ-reactive T cells has been detected, suggesting that adaptive immune responses may exacerbate neuroinflammation and neuronal damage (12). B cells and antibodies also play roles in neurodegenerative diseases. In AD, the role of B cells is less clear, but autoantibodies against neuronal components have been identified, indicating a potential contribution to disease progression (13).

## 2 Inflammasome

Neuroinflammation, characterized by the activation of immune cells and the release of pro-inflammatory mediators, is closely intertwined with inflammasome activation in the CNS. Inflammasomes are multiprotein complexes that include PRRs, adapter proteins where applicable, and inflammatory caspases. They are activated by pathogen-associated molecular patterns (PAMPs) or endogenous danger-associated molecular patterns (DAMPs). Upon activation, PRRs oligomerize, facilitating the binding and oligomerization of the adapter protein apoptosis-associated speck-like protein containing a caspase recruitment domain (ASC). ASC has two death domains (DDs), a pyrin domain (PYD) at the N-terminus and a caspase recruitment domain (CARD) at the C-terminus. ASC oligomerization subsequently activates caspase-1, leading to the maturation and release of the proinflammatory cytokines interleukin-1β (IL-1β) and interleukin-18 (IL-18) (14–16). These cytokines elicit robust inflammatory responses by activating the IL-1β receptor (IL-1βR)/IL-18 receptor (IL-18R)-myeloid differentiation primary response gene 88 (MyD88)-nuclear factor kappa-B (NFκB) pathway. Furthermore, active caspase-1 cleaves gasdermin D (GSDMD), resulting in the liberation of its N-terminus, which forms pores in the plasma membrane (PM) and triggers pyroptosis (14–16). Proper inflammasome activation is crucial for pathogen elimination and the clearance of damaged cell, whereas excessive inflammasomes activity contributes to various diseases, including inflammatory disorders and neurodegenerative diseases.

Based on their cellular localization, PRRs are classified into membrane-bound and cytoplasmic types. TLRs and the C-type lectin receptors (CLRs) are examples of membrane-bound PRRs, whereas NLRs, retinoic acid-inducible gene-I–like receptors (RLRs) and absent in melanoma 2 (AIM2)-like receptors (ALRs) are cytoplasmic PRRs. This review focuses on AIM2, a member of the ALR family, which belongs to the pyrin and hematopoietic, interferon inducible, and nuclear (HIN) domain (PYHIN) family, characterized by a pyrin domain (PYD) at the N-terminus and one or two HIN domains at the C-terminus.

AIM2 is a cytosolic receptor for double-stranded DNA (dsDNA). Previous studies suggested that in the absence of dsDNA, the HIN and PYD domains of AIM2 interact to maintain it in an autoinhibited state. Interaction of dsDNA with the HIN domain alleviates AIM2 from this autoinhibited state (17), promoting the formation of AIM2 PYD filament (18). Nevertheless, Sohn and his colleagues challenged this autoinhibitory model. They proposed that the AIM2 PYD domain is not involved in AIM2 autoinhibition but rather aids in both binding to dsDNA and self-association of AIM2. Additionally, the AIM2 PYD domain enhances the binding of AIM2 to dsDNA, whereas the AIM2 HIN domain’s interaction with dsDNA promotes AIM2 oligomerization (19). Clustering of AIM2 on dsDNA induces filamentous polymerization of AIM2 PYD.

## 3 The mechanisms of AIM2 activation

The HIN domain of AIM2 consists of two oligonucleotide/oligosaccharide binding (OB) folds, each binding to one DNA strand (17, 20, 21). This interaction is predominantly facilitated by electrostatic interactions between positively charged residues of the OB folds and the negatively charged phosphates on the ribose backbone of DNA molecules (17, 20, 21). The binding of AIM2 to dsDNA is sequence-independent due to the lack of interaction between the HIN domain of AIM2 and the DNA bases (22). However, the length of dsDNA is an important factor for efficient activation of the AIM2 inflammasome. A minimum length of 70 base pairs (bp) of dsDNA is required for AIM2 inflammasome activation. However, optimal AIM2 activation requires dsDNA of at least 200 bp in length (17). Moreover, the assembly of the AIM2 inflammasome is expedited by longer dsDNA molecules (23). A recent study suggested that the HIN domain of AIM2 does not exhibit a preference for dsDNA over other nucleic acids. Nonetheless, AIM2 demonstrates a greater affinity for binding to and oligomerizing on dsDNA. Additionally, although AIM2 can assemble filaments on various nucleic acids besides dsDNA, these filaments are poorly organized and fail to enhance ASC polymerization (24).

AIM2 activation has been observed in various pathological conditions, such as neurodegenerative diseases (25, 26), diabetes (27) and heart failure (28). Nevertheless, the mechanisms underlying AIM2 activation in these diseases remain elusive. Emerging evidence suggests that AIM2 can be activated by a variety of types of cytoplasmic DNA, including foreign DNA from virus/bacteria, or self-DNA herniated from the nuclear envelope or mitochondrial membrane in these pathological states.

### 3.1 Bacterial and virus infection

The activation of the AIM2 inflammasome during bacterial infections is primarily attributed to its ability to detect microbial dsDNA. Two essential prerequisites for AIM2 inflammasome activation in the context of bacterial infection are the access of bacteria to the cytoplasm and the subsequent release of bacterial DNA through bacteriolysis. The secretory effector protein SdhA of *L. pneumophila* is known to inhibit the release of bacterial DNA and thereby impede AIM2 inflammasome activation (29). Additionally, infection of macrophages with *F. tularensis* generates cytosolic DNA that specifically binds to AIM2, inducing AIM2 oligomerization, which subsequently leads to the activation of caspase-1 and cell death (30). Peng and colleagues demonstrated that mutations in certain bacterial strains result in increased intracellular lysis, leading to enhanced release of bacterial DNA into the host cell cytosol, thereby triggering AIM2 inflammasome activation (31). Furthermore, a mutation in the lmo2473 gene, which encodes a protein of unknown function, resulted in hyperactivation of the AIM2 inflammasome in *L. monocytogenes* infected macrophages. This hyperactivation was associated with impaired cell wall integrity in the lmo2473 mutant, leading to increased intracellular lysis and consequent DNA release into the cytosol, which in turn augmented AIM2 inflammasome activation (32).

Moreover, AIM2 can recognize cytosolic dsDNA viruses and form an inflammasome complex that enhances host defense by inducing pyroptosis. Classical dsDNA-containing viruses, such as mouse cytomegalovirus (MCMV) and vaccinia virus, are known to activate the AIM2 inflammasome in both mouse bone marrow-derived macrophages (BMDMs) and bone marrow-derived dendritic cells (BMDCs) (33). Human papillomaviruses, which are also dsDNA viruses, specifically infect human keratinocytes and promote the production of cytokines in an AIM2-dependent manner (34). Additionally, siRNA-mediated knockdown of AIM2 in human glomerular mesangial cells infected with hepatitis B virus results in decreased expression of IL-1β, IL-18, and caspase-1 (35).

### 3.2 The herniation of genomic DNA from the nuclear membrane

The herniation of genomic DNA from the nuclear membrane refers to the pathological protrusion or extrusion of nuclear DNA into the cytoplasm, typically resulting from nuclear envelope instability or cellular stress. Nelfinavir, an HIV aspartyl protease inhibitor, has been shown to induce inflammasome formation and trigger IL-1R-dependent inflammation in mice. This drug impairs the maturation of lamin A, a critical structural component of the nuclear envelope, thereby facilitating the release of DNA into the cytosol. Additionally, the absence of AIM2 diminishes nelfinavir-mediated inflammasome activation (36). Furthermore, AIM2 in myoepithelial cells (MECs) from the lacrimal gland can be directly activated by self-genomic DNA (self-gDNA), which might contribute to the pathogenesis of primary Sjögren’s syndrome (pSS) (37).

### 3.3 Mitochondrial dysfunction

Mitochondria dysfunction has emerged as a significant trigger for AIM2 activation. One of the key mechanisms leading to AIM2 activation is the release of mitochondrial DNA (mtDNA) into the cytoplasm. Once in the cytoplasm, mtDNA acts as a potent activator of AIM2 inflammasome. For example, oxidative stress occurring in hepatic steatosis leads to mitochondrial damage, release of mtDNA, and subsequent activation of the AIM2 inflammasome. Additionally, the nonsteroidal anti-inflammatory drug (NSAID)-activated gene-1 (NAG-1)/growth differentiation factor-15 (GDF15) prevents hepatic steatosis by inhibiting the activation of AIM2 (38). Furthermore, cholesterol induces the release of mtDNA and activates AIM2 in macrophages. Studies suggest that oxidized mtDNA activates the NLRP3 inflammasome, whereas non-oxidized mtDNA primarily stimulates the AIM2 inflammasome (39).

## 4 AIM2 protein interactions

Interactions between AIM2 and various protein partners play essential roles in modulating cellular functions beyond inflammasome assembly, ranging from immune regulation to cellular metabolism. These interactions provide insights into the multifaceted roles of AIM2 in neurodegenerative diseases.

**Figure 1:**
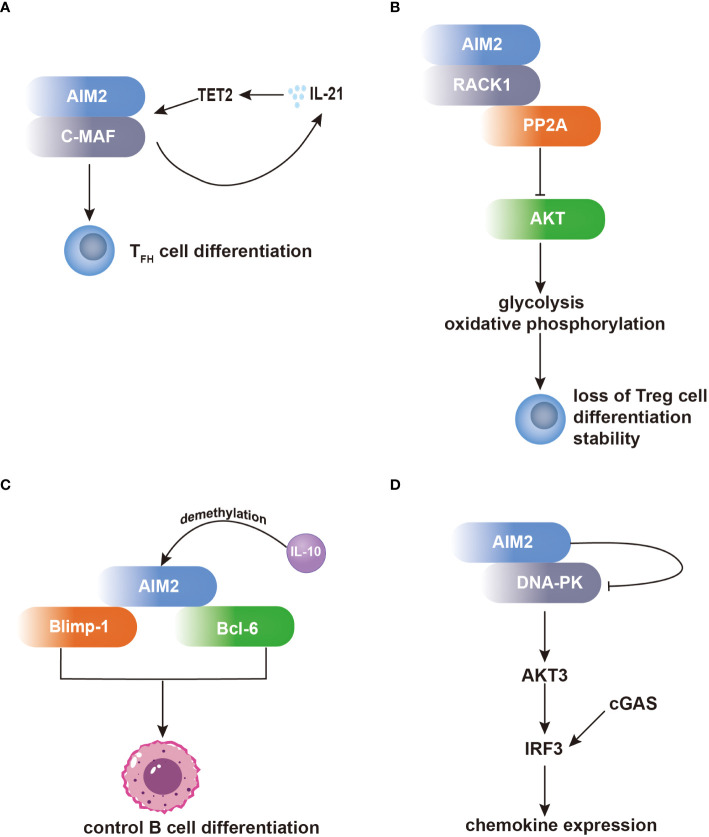
AIM2 modulates the differentiation of T cells and B cells independent of the inflammasome. **(A)** Within T_FH_ cells, AIM2 orchestrates T_FH_ differentiation through c-MAF recruitment, bolstering IL-21 production. Consequently, IL-21 facilitates TET2 recruitment to the AIM2 promoter, enhancing AIM2 transcription. **(B)** Within Treg cells, AIM2 preserves their identity via RACK1 interaction. RACK1 facilitates AKT dephosphorylation by recruiting PP2A, thereby inducing a metabolic shift towards oxidative phosphorylation, crucial for Treg cell stability. **(C)** AIM2 exerts influence over B-cell differentiation by interacting with Blimp-1 and Bcl-6. AIM2’s direct interaction with Bcl-6 upregulates Bcl-6 expression, whereas its binding to Blimp-1 downregulates Blimp-1 expression, impacting B-cell differentiation. Notably, both Blimp-1 and Bcl-6 are transcription factors pivotal in regulating B-cell differentiation. **(D)** Within microglia, AIM2 binds to DNA-PK, effectively suppressing its activity. Consequently, IRF3 phosphorylation is diminished, leading to inhibition of chemokine expression.

One notable set of AIM2 interacting partners are the proteins related to the regulation of T-cell functions. In T follicular helper (T_FH_) cells, AIM2 regulates T_FH_ differentiation by recruiting c-musculoaponeurotic fibrosarcoma (c-MAF). This interaction regulates c-MAF expression, enhances IL-21 production, and recruits hydroxymethyltransferase ten-eleven translocation 2 (TET2) to the AIM2 promoter, leading to DNA demethylation and increased AIM2 transcription (**Figure 1A**) (40). Additionally, AIM2 has been found to modulate the activity of regulatory T (Treg) cells through its interaction with activated protein C kinase 1 (RACK1). In Treg cells, AIM2 preserves Treg cell identity by recruiting RACK1, which induces AKT dephosphorylation by recruiting the phosphatase PP2A. This inhibition of AKT signaling shifts the metabolic program of Treg cells towards oxidative phosphorylation, thereby stabilizing the Treg cell lineage (**Figure 1B**) (41).

Furthermore, AIM2 participates in the regulation of B-cell differentiation through its interactions with transcription factors involved in B-cell fate determination. By directly binding to B lymphocyte-induced maturation protein 1 (Blimp-1) and B-cell lymphoma 6 protein (Bcl-6), AIM2 can modulate their expression levels, thereby influencing the balance between plasma cell differentiation and germinal center reactions. The dynamic interplay between AIM2 and these transcriptional regulators highlights the complexity of B-cell differentiation and the potential regulatory role of AIM2 in antibody production and immune responses (**Figure 1C**) (42).

Additionally, in microglia, AIM2 forms a complex with DNA-dependent protein kinase (DNA-PK), wherein DNA-PK acts as a negative regulator of AIM2-mediated inflammation. Through this interaction, DNA-PK suppresses the activity of AIM2, thereby inhibiting downstream signaling cascades involved in inflammatory gene expression. Specifically, DNA-PK inhibits the activation of AKT3 and subsequent phosphorylation of interferon regulatory factor 3 (IRF3), such effect inhibits cyclic GMP–AMP synthase (cGAS) and DNA-PK comediated-IRF3 activation, leading to the attenuation of microglial antiviral pathway-related inflammation (**Figure 1D**) (43).

**Figure 2:**
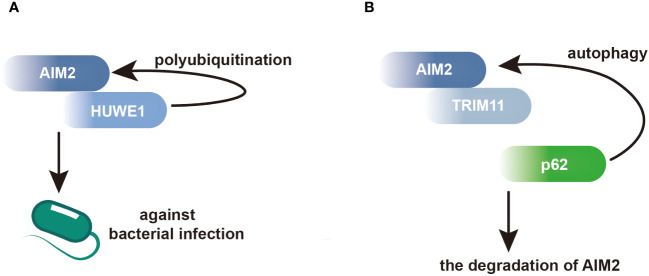
AIM2 modulates its activation and degradation by interacting with its partners in BMDMs. **(A)** Within BMDMs, HUWE1 has been identified as an AIM2-interacting protein, facilitating the polyubiquitination of AIM2 and triggering its activation. This activation enhances host defense against bacterial infections. **(B)** Through its PS domain, TRIM11 interacts with AIM2, facilitating the association between TRIM11 and the autophagic cargo receptor p62. This interaction leads to the degradation of AIM2.

Moreover, AIM2 interacts with proteins involved in the regulation of macrophage activation. In macrophages, the E3 ubiquitin ligase HECT, UBA and WWE domain-containing E3 ubiquitin protein ligase 1 (HUWE1) has been identified as an AIM2-interacting protein. Through its BH3 domain, HUWE1 binds to the HIN domain of AIM2, mediating AIM2 polyubiquitination and promoting its activation (**Figure 2A**) (44). This interaction underscores the importance of post-translational modifications in regulating AIM2 inflammasome activity in macrophages. Additionally, AIM2 interacts with tripartite motif containing 11 (TRIM11), leading to AIM2 auto-polyubiquitination and subsequent degradation via selective autophagy. This regulatory mechanism serves as a feedback loop to control AIM2 levels and prevent excessive inflammasome activation (**Figure 2B**) (45).

## 5 Activation and roles of AIM2 inflammasome in different cells

### 5.1 Neurons

In recent years, researchers have consistently observed high expression of the AIM2 inflammasome in neurons across various CNS diseases (46, 47). Upon activation, the AIM2 inflammasome secretes caspase-1-cleaving inflammatory cytokines in neurons, ultimately inducing neuronal cell death via pannexin-1 channel opening (48). Additionally, the inhibition of pannexin-1 channels using probenecid in a rat model of subarachnoid hemorrhage reduced AIM2 inflammasome activation in neurons, resulting in the attenuation of brain edema and neurological deficits in rats (49). Furthermore, adding synthetic dsDNA to cultured neurons induces IL-1β secretion in an AIM2-dependent manner, which consequently enhances axon extension. Behavioral analyses further reveal that AIM2 deletion in mice induces anxious behaviors, and impairs locomotion and auditory fear memory (46).

### 5.2 Dendritic cells

In DCs, AIM2 plays a pivotal role in orchestrating immune responses against various pathogens and in shaping adaptive immunity. DCs are professional antigen-presenting cells required for initiating and modulating immune responses. Upon encountering pathogens, DCs recognize and capture antigens, process them, and present antigenic peptides to T cells, thereby activating adaptive immune responses. Studies have shown that the activation of AIM2 inflammasome in DCs is important for mounting effective immune responses against various pathogens, including bacteria, viruses, and parasites. For example, AIM2 activation in DCs is essential for controlling infections caused by pathogens such as *Francisella novicida, Mycobacterial species and Adenovirus* (50).

In addition, AIM2 activation in DCs has been implicated in cancer immunotherapy. The activation of AIM2 contributes to the formation of an immunosuppressive microenvironment within melanoma. Vaccination with AIM2-deficient DCs enhances the therapeutic efficacy of adoptive T-cell therapy and anti–programmed death-ligand 1 (PD-L1) immunotherapy in tumors unresponsive to current treatments. Similarly, mouse DCs treated with AIM2 siRNA *in vivo* and human DCs *in vitro* exhibit potent anti-tumor immune responses (51).

Moreover, differentiation of monocytes into DCs induced by CD137 ligand (CD137L) activates the AIM2 inflammasome, leading to the polarization of CD8+ T cells towards a cytotoxic T lymphocyte (Tc1) phenotype. This conversion enhances T-cell response against cancer‐associated viruses (52). Additionally, activation of AIM2 in plasmacytoid DCs induces Ca^2+^ release, triggering calpain activation and IL-1α release, thereby promoting tumor cell proliferation in the lung (53).

### 5.3 Monocyte

Monocytes can be categorized into two groups. One group consists of immature proinflammatory monocytes, which express CD115, lymphocyte antigen 6C (Ly6C), and C-C chemokine receptor type 2 (CCR2) on their cell membrane. Upon activation, these monocytes migrate to injury sites and differentiate into proinflammatory macrophages. The other group consists of regulatory/patrolling monocytes, which express low levels of Ly6C and high levels of CX3CR1, and do not express CCR2. Under healthy conditions, these monocytes differentiate into tissue-resident macrophages.

AIM2 activation in monocytes contributes to T cell death following tissue injuries, increasing susceptibility to life-threatening infections. Monocytes detect DNA released during injury via the AIM2 inflammasome, upregulating Fas ligand (FasL) expression and inducing apoptosis in extrinsic T cells (54). Furthermore, Infection of monocytes with severe acute respiratory syndrome-coronavirus-2 (SARS‐CoV‐2) triggers inflammatory responses via activation of the AIM2 inflammasome, thereby contributing to the pathogenesis of COVID-19 (55).

### 5.4 Macrophages/Microglia

Macrophages exhibit diverse functions including pathogen defense and wound healing. They are classified into “pro-inflammatory (M1)” and “anti-inflammatory (M2)” macrophages based on their activation conditions. Initially, this dichotomy was proposed to simplify the understanding of macrophage functions in various physiological and pathological contexts. However, recent research suggests that macrophages exhibit a much broader spectrum of activation states, influenced by the complex interplay of signals within their microenvironment. This continuum of activation challenges the binary classification, as macrophages can simultaneously express markers and functions characteristic of both M1 and M2 phenotypes or transition between states in response to dynamic changes in their environment. Consequently, the simplistic M1/M2 paradigm may not adequately capture the full functional diversity and plasticity of macrophages (56).

Considerable evidence supports the role of AIM2 activation in eliminating intracellular bacteria and DNA viruses within infected macrophages, such as *Francisella tularensis, Listeria monocytogenes, and Murine cytomegalovirus* (30, 33). AIM2 activation in macrophages also contributes to kidney injury, and renal carcinoma. In a mouse model of kidney injury induced by unilateral ureteric obstruction (UUO), the activation of AIM2 inflammasome in macrophages, triggered by DNA uptake, regulates renal inflammation and fibrosis (57). In renal carcinoma models, AIM2 enhances the polarization switch of tumor-associated macrophages (TAMs) from the anti-inflammatory M2 phenotype to the pro-inflammatory M1 phenotype (58). Additionally, AIM2 collaborates with adrenergic signaling, promoting macrophages to assume an immunosuppressive phenotype by increasing the expression of programmed death-ligand 1 (PD-L1) and indoleamine 2,3-dioxygenase (IDO). blocking either the AIM2 inflammasome or α1-adrenergic receptor (α1-AR) diminishes IL-1β release triggered by chimeric antigen receptor T-cell (CAR-T) therapy (59).

Microglia, often termed the “sentinels of the brain,” are resident immune cells of the CNS crucial for maintaining its homeostasis. Microglial AIM2 functions have also been investigated. In the 1-methyl-4-phenyl-1,2,3,6-tetrahydropyridine (MPTP) mouse model, microglial AIM2 activation diminishes cGAS - mediated antiviral inflammation. This inhibition occurs independently of the inflammasome and involves IRF3 phosphorylation suppression (25). Microglial AIM2 deficiency worsened behavioral and pathological symptoms in both MPTP-induced and transgenic PD mouse models (25). Additionally, microglial AIM2 alleviates symptoms of experimental autoimmune encephalomyelitis (EAE) independent of inflammasome activation. Absence of AIM2 enhances microglial activities and peripheral immune cell infiltration into the CNS, exacerbating neuroinflammation and demyelination during disease progression (43). Furthermore, microglial AIM2 contributes to age-related cognitive dysfunction by modulating complement-dependent microglial phagocytosis (60). Similarly, microglia exhibit heightened expression levels of AIM2 in AD mice. However, cognitive and synaptic dysfunction in AD mice were ameliorated upon conditional knockout of microglial AIM2. This knockout also led to a reduction in excessive microglial phagocytosis of synapses via the inhibition of complement activation (61).

### 5.5 B cells

B cells function in antibody production, antigen presentation and cytokine production. Antibody binding to targets can trigger antibody-dependent cellular cytotoxicity (ADCC) and complement-dependent cytotoxicity (CDC). Moreover, B cells perform antigen presentation by internalizing, processing and presenting antigens to CD4+ T cells. Additionally, B cells secrete both pro-inflammatory and anti-inflammatory cytokines in response to stimulation.

AIM2 expression is confined to mature B cells, specifically within the CD27 positive subset. In response to IFN-γ, these cells upregulate AIM2 mRNA expression, while activation of B-cell receptor by CD40 leads to downregulation of AIM2, enabling B-cell proliferation (62). AIM2 in B cells has been found to play a noncanonical role in regulating CXCL16 production. AIM2 knockout enhances CXCL16 production in B cells while reducing the expression of homing receptors [sphingosine-1-phosphate receptor 1 (S1PR1) and CD62 ligand (CD62L)] in CD8+ T cells, thereby increasing their infiltration and retention in chronic inflammatory tissues independently of the inflammasome (63). Additionally, AIM2 deficiency in B cells modulates B-cell differentiation through the Blimp-1-Bcl-6 axis, leading to the amelioration of systemic lupus erythematosus (SLE) (42).

### 5.6 T cells

T cells encompass helper, regulatory, cytotoxic and memory subsets. Helper T cells secrete cytokines to facilitate B cell differentiation upon stimulation. Regulatory T cells modulate immune responses. Cytotoxic T cells eliminate infected cells. Memory T cells, specific to antigens, swiftly transition into effector T cells upon re-exposed to the antigen.

AIM2 plays a cell-intrinsic role in CD4+ T-cell differentiation. AIM2 induces the differentiation of CD4+ T cells into FOXP3+ regulatory T cells (Treg). RNA sequencing reveals that Treg differentiation is not primarily driven by the expression of signature genes, but rather by the modulation of cell metabolism. Similarly, in a T-cell transfer model of colitis, T cells deficient in ATM2 preferentially differentiated into FOXP3+ cells *in vivo* (64). Moreover, AIM2 enhances Treg cell stability and protects mice from autoimmune encephalomyelitis and inflammatory colitis. Transcription factors associated with Treg cells, including RUNX1, ETS1, BCL11B and CREB can occupy AIM2 promoter. Moreover, AIM2 mitigates EAE symptoms by promoting the switch from glycolysis to oxidative phosphorylation in Treg cells (41). Additionally, AIM2 influences the development of SLE by regulating T_FH_ cell differentiation through IL-21-induced TET2 recruitment and the interaction between AIM2 and c-MAF (40).

## 6 The roles of AIM2 in neurodegenerative diseases

### 6.1 AD

AD is the most prevalent neurodegenerative disease. While amyloid-β (Aβ) deposition in the brain has been proposed as the initiating factor in AD, accumulating evidence challenges this hypothesis. Conversely, elevated levels of inflammatory markers in AD patients and the identification of AD risk genes associated with innate immune functions imply that neuroinflammation may play a role in AD pathogenesis.

In AD, Aβ is generated through the sequential cleavage of two enzymes and cleared from the brain via transport into the cerebrospinal fluid (CSF). Upon surpassing a critical concentration threshold, Aβ aggregates into oligomers and fibrils, serving as DAMPs that trigger inflammasome activation. Research has demonstrated that Aβ can activate the AIM2 inflammasome in BV2 cells and primary cultured microglia, leading to increased mRNA and protein expression of AIM2 (65).

Numerous studies have demonstrated the implication of AIM2 in AD (**Table 1**). Deletion AIM2 reduces Aβ deposition and suppresses microglial activation in 5XFAD mice; However, it does not improve the memory and anxiety phenotypes of these mice. Interestingly, the knockout of AIM2 in 5XFAD mice leads to an increase in inflammatory cytokine expression (26). Conversely, deleting AIM2 in APP/PS1 mice improves spatial memory, promotes the induction of long-term potentiation (LTP) in hippocampal slices, and induces structural changes in dendrites. Transcriptional microarray analysis revealed that AIM2 deficiency in APP/PS1 mice alters the expression levels of various proteins, including Pten, Homer1, and Ppp2r3a (66). Moreover, ectopic expressing optineurin (OPTN) inhibits the activation of AIM2 inflammasomes induced by Aβ oligomers in APP/PS1 mice (65). Furthermore, AIM2 regulates microglial phagocytosis of synapses, triggering activation of the complement system through the classical pathway, ultimately contributing to cognitive impairment in Aβ1–42-induced AD mice (61).

**Table 1:** An overview of studies on the AIM2 inflammasome in neurodegenerative diseases.

Disease Type	Animal models/Patients	Treatments	Therapeutic effects	References
AD				
AD	5XFAD mice	AIM2–/–	mitigates Aβ deposition and microglial activation increases in inflammatory cytokine expression	Wu et al., (26)
AD	APP/PS1 mice	AIM2–/–	improves spatial memory, promotes LTP induction induces structural changes in dendrites	Chen et al., (66)
AD	APP/PS1 mice	optineurin	deactivates microglial cells and astrocytes reduces neuroinflammation	Cao et al., (65)
AD	Injection of Aβ1–42 into the bilateral hippocampal CA1	AIM2 overexpression lentivirus and conditional AIM2-/-	decreases microglial phagocytosis of synapses, reduces the activation of the complement system	Ye et al., (60)
PD				
PD	LPS induced PD model	Crocin	reduces the SN’s LPS-induced neuroinflammation and DA neuronal loss, decreases cell infiltration	Alizadehmoghaddam et al., (67)
PD	MPTP-induced PD model; A30P PD model	conditional AIM2-/-	exacerbates PD symptoms and neuroinflammation independent of the inflammasome	Rui et al., (25)
TBI				
TBI	Culture neurons: TBI patients	dA:dT	Treatment with Poly(dA:dT) triggers ASC speck formation and IL-1β release; CSF from TBI patients can induce AIM2 activation and neuronal pyroptosis	Adamczak et al., (48)
TBI	CCI mouse model	Ac-YVAD-cmk	mitigates TBI-induced brain edema and BBB leakage	Ge et al., (68)
TBI	CCI mouse model	VX765	attenuates pyroptosis and inhibits the HMGB1/TLR4/NF-κB pathway	Sun et al., (69)
SCI	contusive SCI rat model	melatonin and 17β-estradiol	decreases AIM2 protein expression and signals. associated with NLR synthesis and activation	Majidpoor et al., (70)
TSCI	Mouse TSCI model	miR-672–5p	repairs SCI-induced damage, inhibits neuronal pyroptosis, promotes the recovery of functional behavior in TSCI mice	Zhou et al., (71)
SCI	rat cervical SCI model	rapamycin	transforms microglia to M2 phenotype, improves the outcome of cervical SCI	Xiao et al., (72)
SCI	rat SCI model	SC treatment	promotes neuronal survival and myelination, reduces the expression of inflammasome components	Kharazinejad et al., (73)
SCI	mouse SCI model	RO27‐3225	improves functional recovery, inhibits AIM2 activation, maintains mitochondrial homeostasis, represses oxidative stress, and prevents Drp1 translocation to the mitochondria.	Wang et al., (74)

### 6.2 PD

PD ranks as the second most common neurodegenerative disease, characterized by the gradual loss of dopaminergic neurons in the substantia nigra. Numerous studies have implicated neuroinflammation in the pathogenesis of PD (75, 76). Limited research has investigated the role of AIM2 in PD (**Table 1**). In MPTP-induced mouse models, the NLRP3 inflammasome exhibits significant activation in the nigrostriatal system, while the activities of AIM2 and other inflammasomes remain unaltered (77). Conversely, a separate study has shown that in the MPTP mouse model, microglial AIM2 exerts a negative regulatory effect on neuroinflammation independent of the inflammasome, and its deficiency exacerbates symptoms in both MPTP-induced and transgenic PD mouse models (25). Furthermore, crocin, one of herbal medicines, significantly reduced neuroinflammation and the loss of DA neurons in the SN of lipopolysaccharide (LPS)-induced PD models. Hematoxylin and eosin (H&E) staining revealed that crocin treatment reduced cell infiltration at the site of LPS injection, potentially through the inhibition of AIM2 expression (67).

### 6.3 TBI/SCI induced neurodegeneration

TBI and spinal cord injury (SCI) are significant causes of neurodegeneration, with neuroinflammation playing a central role in this process. Following TBI or SCI, there is an immediate activation of the innate immune response, characterized by the rapid infiltration of immune cells such as microglia and astrocytes to the site of injury. These cells release pro-inflammatory cytokines, chemokines, and ROS, which exacerbate neuronal damage. This sustained neuroinflammation not only contributes to secondary injury mechanisms, including excitotoxicity, oxidative stress, and blood-brain barrier (BBB) disruption, but also impedes the regenerative processes essential for neural recovery (78, 79). The interplay between neuroinflammation and neurodegeneration creates a detrimental feedback loop, where ongoing inflammation perpetuates neuronal loss and synaptic dysfunction, ultimately leading to progressive cognitive and motor deficits observed in TBI and SCI patients (78, 80).

Numerous studies indicate that AIM2 activation is crucial in TBI pathology (**Table 1**). Treatment with Poly(dA:dT) triggers ASC speck formation and IL-1β release in embryonic cortical neurons. Moreover, CSF from TBI patients can induce AIM2 activation and neuronal pyroptosis *in vitro* (48). In the controlled cortical impact (CCI) mouse model, brain microvascular endothelial cells (BMVECs) extracted from the injured cerebral cortex exhibit activation of both NLRs and AIM2 inflammasomes. Suppressing pyroptosis with Ac-YVAD-cmk mitigates TBI-induced brain edema and BBB leakage (68). Furthermore, TBI mice show elevated expression of NLRs and the AIM2 inflammasome in the cerebral cortex. VX765, a selective caspase-1 inhibitor, attenuates pyroptosis and inhibits the HMGB1/TLR4/NF-κB pathway (69). A recent study has discovered that AIM2 can serve as a serum marker reflecting the severity of human severe TBI and predicting poor outcomes (81).

Furthermore, in the healthy spinal cord, AIM2 expression is predominant in neurons, astrocytes, microglia, and oligodendrocytes, its expression increases following SCI (82). Additionally, in rat SCI models, both NLRs and AIM2 become activated 72 h post-injury. Hormonal therapy decreases AIM2 protein expression and signals associated with NLR synthesis and activation (70). Moreover, miR-672–5p, a crucial miRNA found in anti-inflammatory microglial exosomes, targets AIM2 gene expression. Applying miR-672–5p can repair SCI-induced damage (71).

In addition, pre-treatment with rapamycin has been demonstrated to ameliorate the outcome of cervical SCI in rats by modulating the AIM2 signaling pathway (72). Meanwhile, Schwann cell (SC) therapy has exhibited greater efficacy in dampening AIM2 inflammasome activation and the associated inflammatory pathway in SCI compared to Wharton’s jelly mesenchymal stem cells (WJ-MSCs) (73). Additionally, melanocortin receptor 4 (MC4R) has shown a protective effect on neurons by inhibiting AIM2 activation post-SCI, partly attributed to its regulation of mitochondrial homeostasis (74).

## 7 Targeting of AIM2 signaling pathways as a novel therapeutic approach in neurodegenerative diseases

**Figure 3:**
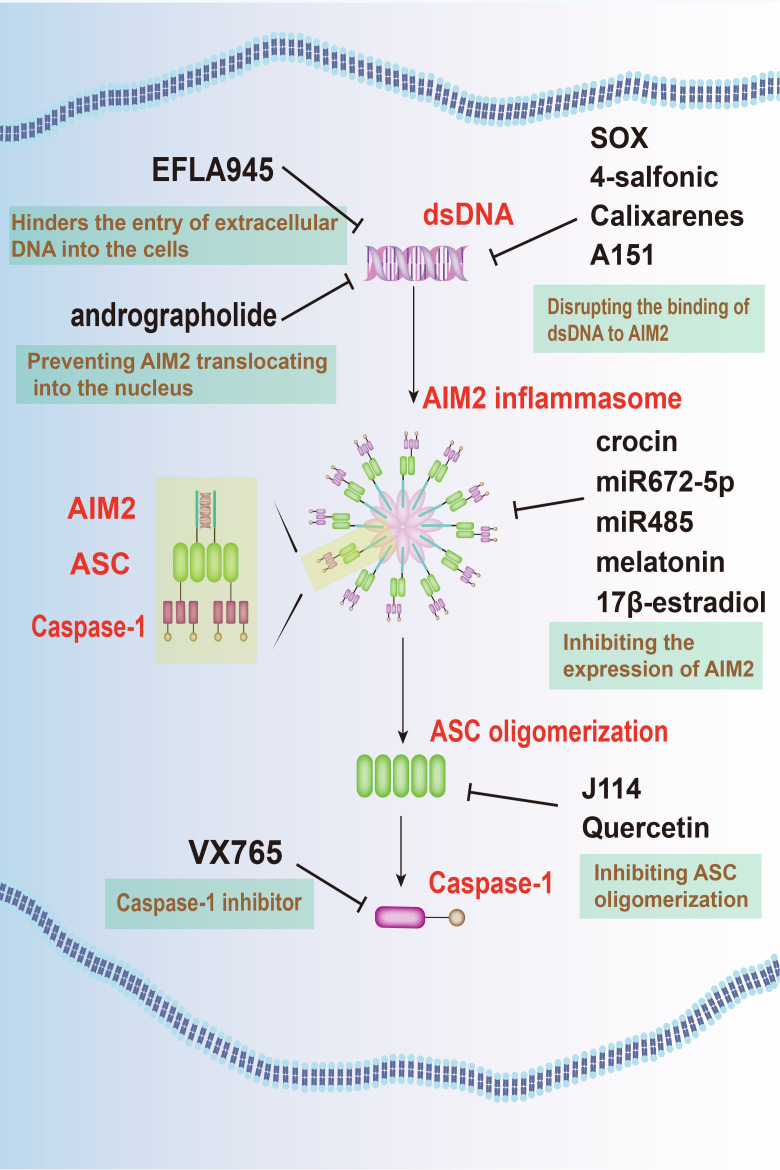
Targeting AIM2 signaling pathways offers a novel therapeutic approach for neurodegenerative diseases. Several agents, such as EFLA945, andrographolide, KSHV-encoded SOX protein, 4-sulfonic calixarenes, and A151, have been identified to inhibit AIM2 activation. Additionally, crocin, miRNAs, along with substances like melatonin and E2, are effective in inhibiting the expression of AIM2. Furthermore, J114 and quercetin are known to hinder ASC oligomerization, while VX-765 prevents the activation of caspase-1. These combined approaches offer a multi-faceted strategy to mitigate the detrimental effects of AIM2 in neurodegenerative conditions.

Targeting of AIM2 signaling pathways presents a promising therapeutic avenue for neuroprotection in neurodegenerative diseases, with various strategies focusing on regulating AIM2 expression, activation, and downstream signaling pathways (**Figure 3**).

### 7.1 Inhibiting AIM2 activation

One such approach involves inhibiting AIM2 activation by targeting its upstream regulators. Agent like EFLA945 hinders the entry of extracellular DNA into the cells, thus preventing AIM2 activation and protecting against cell damage (83). In addition, andrographolide, an active component extracted from *andrographis paniculate*, effectively prevented AIM2 activation by preventing it from translocating into the nucleus to sense DNA damage. By this way, it ameliorates radiation-induced lung injury (RILI) (84). Furthermore, compounds like the kaposi’s sarcoma-associated herpesvirus (KSHV)-encoded SOX protein (85), 4-sulfonic calixarenes (86), and A151 (87) disrupt the binding of dsDNA to AIM2, inhibiting AIM2 inflammasome activation and mitigating inflammatory responses.

### 7.2 Inhibiting AIM2 expression

An alternative approach focuses on the inhibition of AIM2 expression within the CNS. Techniques such as microRNAs (miRNAs), along with substances like melatonin and 17β-estradiol (E2), offers targeted suppression of AIM2, potentially mitigating neuroinflammatory responses.

MiRNAs, found ubiquitously in organisms, possess the capability to selectively bind to target mRNA molecules, thereby regulating gene expression post-transcriptionally by inducing their degradation or inhibiting translation. MiR672–5p downregulates AIM2 expression, inhibiting neuronal pyroptosis via the AIM2/ASC/caspase-1 pathway (71). Similarly, miR-485 suppresses AIM2 expression by directly interacting with its 3’-UTR. Long non-coding RNA (lncRNA) maternally expressed gene 3 (MEG3) acts as a sponge for miR-485, ultimately leading to pyroptosis and inflammation during cerebral ischemia/reperfusion (I/R) mediated by AIM2 (88).

Moreover, hormonal therapy employing melatonin and E2 presents significant promise in addressing CNS injuries. Melatonin, primarily secreted by the pineal gland, confers protective effects against neuronal injury, while E2 demonstrates substantial anti-inflammatory properties. Research suggests that both melatonin and E2 reduce the upregulation of AIM2 expression, providing potential therapeutic pathways for neurodegenerative conditions (70, 89). In addition, crocin, an herbal medicine, also attenuates the LPS-induced neuroinflammation in PD by inhibiting AIM2 expression (67).

### 7.3 Inhibiting downstream signaling pathways of AIM2

Promising drug candidates targeting AIM2 signaling pathways have also emerged for potential clinical applications in neurodegenerative disease treatment. VX-765, for instance, acts as a selective caspase-1 inhibitor, effectively halting downstream inflammatory responses triggered by AIM2 inflammasome activation. Preclinical studies have confirmed VX-765’s efficacy in alleviating neuroinflammation and neuronal damage in animal models of TBI (69).

On the other hand, J114 disrupts the interaction between AIM2 and the adaptor protein ASC, hindering ASC oligomerization. This disruption leads to significant inhibitory effects on caspase-1 activation and IL-1β release in human THP-1 macrophages (90). Quercetin, a dietary flavonol abundant in various natural sources, stands out among polyphenols due to its potent antioxidant properties demonstrated in numerous studies. It interferes with ASC oligomerization, thereby impeding AIM2 inflammasome activation and preventing IL-1-mediated mouse vasculitis (91).

## 8 The limitations of current research on AIM2 and the challenges faced in therapeutic development

Despite significant advances in understanding the role of AIM2 in neuroinflammation and neurodegenerative diseases, several limitations and challenges persist in the current research landscape. One major limitation is the complexity of the AIM2 signaling pathways and their interactions with various cellular processes. AIM2 is known to modulate the differentiation and function of immune cells such as T cells, B cells, and microglia through both inflammasome-dependent and independent mechanisms. However, the precise molecular mechanisms underlying these interactions are not fully elucidated. This gap in knowledge hampers the development of targeted therapies, as the intricate network of signaling pathways must be thoroughly understood to design effective interventions.

Furthermore, the heterogeneity of neurodegenerative diseases adds another layer of complexity to AIM2 research. Diseases such as AD and PD exhibit diverse pathological features and progression patterns, influenced by genetic, environmental, and lifestyle factors. This heterogeneity complicates the identification of universal therapeutic targets within the AIM2 pathway. Additionally, patient-specific variations in AIM2 expression and activity further challenge the development of standardized treatments. Personalized medicine approaches, which tailor treatments based on individual patient profiles, may offer a solution but require extensive research to identify relevant biomarkers and develop corresponding therapeutic strategies.

In the context of therapeutic development, another critical issue is the delivery and specificity of AIM2-targeted drugs. Ensuring that these therapies can cross the BBB and reach affected regions in the brain at therapeutic concentrations is a significant challenge. Moreover, achieving specificity in targeting AIM2 without affecting other inflammasome pathways or immune functions is crucial to minimize potential side effects and off-target effects. Current therapeutic candidates, such as small molecule inhibitors and biologic agents, show promise in preclinical models, but their efficacy and safety in humans remain to be rigorously tested.

## 9 Conclusion

The investigation of AIM2 signaling in neurodegenerative diseases has unveiled its multifaceted roles in various pathological conditions. Its involvement in diverse cellular processes underscores its potential as a therapeutic target in these diseases. Several drug candidates targeting AIM2 signaling pathways offer promise for clinical applications in the treatment of neurodegenerative diseases. These candidates have demonstrated efficacy in preclinical models. However, further research is warranted to elucidate the precise mechanisms underlying AIM2-mediated neuroinflammation and its implications for therapeutic development.

## Author contributions

KY: Conceptualization, Supervision, Writing – original draft. XW: Writing – original draft. HP: Writing – original draft. XQW: Writing – review & editing. YH: Writing – review & editing. YY: Writing – review & editing. XZ: Visualization, Writing – review & editing. TS: Conceptualization, Funding acquisition, Supervision, Writing – original draft.
